# Transition from child and adolescent mental health services to adult mental health services

**DOI:** 10.1192/bjo.2021.910

**Published:** 2021-06-18

**Authors:** Tania Saour

**Affiliations:** Central North West and West London Mental Health Trust

## Abstract

**Background:**

The prevalence and recognition of mental health conditions in young people is growing. Around 50% of lifetime mental illness (except dementia) begins by the age of 14. Around 75% of adults requiring secondary mental health services developed problems prior to 18.

The TRACK study of young people's transitions from CAMHS to AMHS has found that up to a third of teenagers are lost from care during transition and a further third experience an interruption in their care.

A CQUIN for Transition has concluded that young children should have a transition plan 6 months before they turn 18.

**Method:**

All young people aged 17 and a half years old were included in the data collection for this audit. Clinical information was reviewed using the West London RIO computer system. While reviewing the clinical documentation I was recording whether:

Transitional plans had been discussed with the young person.

If yes, what were they?

Had a referral been made to the appropriate service?

**Result:**

There were 180 open cases to the Hounslow Adolescent Team. 35 cases were over 18:

At least 16 of these cases needed to be closed as no intervention was being provided.

14 cases had an unclear plan.

Of the 25 cases aged between 17.5 and 18 years of age transitional plans were:

Transition was discussed in 11 cases (44%). This meant that transitional plans were not discussed in 56% of young people.

Of these 11 cases 7 referrals were completed. (28%)

**Conclusion:**

The lack of consistent protocols for transition remains a significant barrier to health care provided to young people.

Transitional planning needs to take place in an effective and timely manner to ensure continued patient centred care.

Transitional discussions to be made a regular agenda item at team meetings.

Care co-ordinator to be informed and reminded that transitional plans need to be explored with young people.

Following a re-audit of this data 6 months on 100% of cases over the age of 18 were closed and transition was discussed in the remaining 56%.

